# The *waaL* gene mutation compromised the inhabitation of *Enterobacter* sp. Ag1 in the mosquito gut environment

**DOI:** 10.1186/s13071-015-1049-1

**Published:** 2015-08-27

**Authors:** Dong Pei, Jinjin Jiang, Wanqin Yu, Phanidhar Kukutla, Alejandro Uentillie, Jiannong Xu

**Affiliations:** Biology Department, New Mexico State University, Las Cruces, NM 88003 USA; Entomology Department, Cornell University, Isaac, NY USA

**Keywords:** *Enterobacter* sp. Ag1, Lipopolysaccharide, *waaL* gene, O antigen ligase, Inhabitation, Oxidative stress, Mosquito gut, Malaria mosquito

## Abstract

**Background:**

The mosquito gut harbors a variety of bacteria that are dynamically associated with mosquitoes in various contexts. However, little is known about bacterial factors that affect bacterial inhabitation in the gut microbial community. *Enterobacter* sp. Ag1 is a predominant Gram negative bacterium in the mosquito midgut.

**Methods:**

In a mutant library that was generated using transposon Tn5-mediated mutagenesis, a mutant was identified, in which the gene *waaL* was disrupted by the Tn5 insertion. The *waaL* encodes O antigen ligase, which is required for the attachment of O antigen to the outer core oligosaccharide of the lipopolysaccharide (LPS).

**Results:**

The *waaL*^−^ mutation caused the O antigen repeat missing in the LPS. The normal LPS structure was restored when the mutant was complemented with a plasmid containing *waaL* gene. The *waaL*^−^ mutation did not affect bacterial proliferation in LB culture, the mutant cells grew at a rate the same as the wildtype (wt) cells. However, when *waaL*^−^ strain were co-cultured with the wt strain or complemented strain, the mutant cells proliferated with a slower rate, indicating that the mutants were less competitive than wt cells in a community setting. Similarly, in a co-feeding assay, when fluorescently tagged wt strain and *waaL*^−^ strain were orally co-introduced into the gut of *Anopheles stephensi* mosquitoes, the mutant cells were less prevalent in both sugar-fed and blood-fed guts. The data suggest that the mutation compromised the bacterial inhabitation in the gut community. Besides, the mutant was more sensitive to oxidative stress, demonstrated by lower survival rate upon exposure to 20 mM H_2_O_2_.

**Conclusion:**

Lack of the O antigen structure in LPS of *Enterobacter* compromised the effective growth in co-culture and co-feeding assays. In addition, O-antigen was involved in protection against oxidative stress. The findings suggest that intact LPS is crucial for the bacteria to steadily stay in the gut microbial community.

## Background

Vector mosquitoes are responsible for the transmission of deadly diseases, such as malaria and Dengue fever, which poses serious threats to human health and severe burdens to socioeconomic development. Mosquito control is one of the effective intervention strategies to interrupt transmission of mosquito-borne diseases. A dynamic microbial community resides in the mosquito gut ecosystem [1–10]. Many efforts have been made in characterizing taxonomic structure of mosquito gut microbiome. The core bacterial inhabitants have been identified in the phyla Proteobacteria, Bacteroidetes, Actinobacteria and Firmicute. Predominant taxa belong to the families Enterobacteriaceae, Acetobacteraceae, Pseudomonadaceae and Flavobacteriaceae [3, 7, 11, 12]. The microbes have significant impacts on various mosquito life traits, such as development, reproduction, fecundity, immunity and vector competence [6, 9, 11, 13–23]. For example, *Asaia* sp. SF2.1 has been shown to be able to accelerate the larval development of *Anopheles gambiae* when introduced into the aquatic habitats where the mosquito larvae grew [17]. On the opposite, the depletion of *Asaia* by antibiotic rifampicin would cause delay of the larval development in *An. stephensi* [18]. The presence of native gut bacteria is essential for maintaining an effective basal immunity that determines the susceptibility to malaria in both *An. gambiae* [20, 22] and *An. stephensi* [14, 24]. Apparently, a symbiotic relationship between the gut microbiome and the mosquito hosts largely depends on a stably associated core microbial community.

The structure of gut microbial communities varies upon gut conditions of diet types, i.e., sugar meals or blood meals [7, 21]. The sugar-fed gut is carbohydrate-rich while the blood-fed gut is protein-rich environment. Previously, we have shown that the microbial composition shifts upon diet changes. In the sugar-fed gut, especially before the first blood meal is taken, the taxonomic composition is more complicated than that in the blood-fed gut. After taking blood meal, taxonomic complexity drops with the enrichment of enteric bacteria [7]. In wild population, the gut bacterial composition varies among individuals [22]. So far, most studies have focused on the taxonomic composition and community structural fluctuations in different stages of the life history. However, little is known about what bacterial factors affect bacteria to establish in a complex microbial community in the gut environment.

Bacteria in the genus *Enterobacter* were often found in the mosquito guts [2, 3, 7, 16], and in the blood-fed gut *Enterobacter* was one of the enteric bacteria that expanded following blood ingestion [7]. Therefore *Enterobacter* would be a good representative for studying how bacteria reside in the gut. In an effort to recognize bacterial factors that are involved in the gut inhabitation, we have isolated a strain of *Enterobacter* sp. from the gut of a mosquito lab colony [25]. In this study, we generated a Tn5 mediated mutant library of *Enterobacter* sp. and characterized a mutant, *waaL*^−^. The *waaL* encodes lipopolysaccharide (LPS) O antigen ligase. The *waaL* mutation caused the lack of O antigen in the LPS and the increased sensitivity to hydrogen peroxide. In addition, the mutant bacteria were compromised in competing with the wild type bacteria *in vitro* in the co-culture and *in vivo* in the mosquito gut, suggesting that the O antigen associated LPS structure is required for the bacteria to reside in a microbial community in the mosquito gut.

## Methods

### Bacterial strains and mutagenesis

*Enterobacter* sp. Ag1 was originally isolated from the gut of mosquito *Anopheles gambiae* [25]. A mutant library was generated using EZ-Tn5™ < R6Kγ*ori*/KAN-2 >Tnp Transposome kit (Epicentre, WI). First, the *Enterobacter* cells were grown in Luria-Bertani (LB) broth at 28 °C to the exponential phase (OD_600nm_ = 0.7). Cells were harvested by centrifugation at 5000 × g for 5 min and washed twice with 100 mL ice-cold H_2_O and twice 50 mL ice-cold 10 % glycerol. Cells were finally suspended in 1 mL of 10 % ice-cold glycerol, and 100 μL aliquot were used for electroporation. A mutant library was prepared following manufacturer’s instructions. In brief, 1 μL of Tnp transposome was added to the electrocompetent cells, and electroporation (5 ms, 12.5 kV) was performed using an Eporator (Eppendorf, NY). Electroporated cells were immediately recovered by adding 1 mL of SOC broth and incubated at 28 °C for 2 h. The transformants were plated on LB with 40 μg/mL kanamycin (KAN) and incubated at 28 °C overnight. The colonies were further processed for screening genes with a Tn5 insertion. The defense capability against anti-oxidative stress was speculated to be associated with the expansion of enteric bacteria in the blood-fed mosquito gut [7]. The wt *Enterobacter* cells were first examined for the sensitivity to paraquat (Sigma-Aldrich), an oxidative stressor. The bacterial growth was measured in the presence of paraquat at a series of concentration 0, 2, 4, 6, 8 mM (unpublished data). The growth inhibitory effect was dose dependent. In the presence of 6 mM paraquat, the bacteria were able to grow but with a reduced growth rate (unpublished data). Therefore, 6 mM paraquat was selected for screening mutants. Single transposed colonies from LB-KAN plates were inoculated in the LB broth with both 40 μg/mL KAN and 6 mM paraquat. The OD_600nm_ was measured hourly for 5 h. The wt cells were used as control. The transformants that had a growth rate slower than the wt were harvested for gene identification.

### Identification of disrupted genes

To identify the genes having a Tn5 insertion, genomic DNA was isolated from the mutant cells using DNAzol reagent (Invitrogen, NY) and digested with *Eco*RI (NEB, MA), which has no cutting site in the Tn5. Subsequently, the digested DNA was purified, and the *Eco*RI ends were ligated with the T4 DNA Ligase (NEB, MA). The ligation was transformed into electrocompetent EC100D pir-116 *E. coli* (Epicentre, WI) by electroporation, following the manufacturer’s instruction. The transformants were recovered on the LB agar plates with 40 μg/mL KAN overnight at 37 °C. Colony PCR was performed to confirm the presence of R6Kγ*ori and* KAN-2 using the primers provided in the kit. Plasmids were extracted from positive colonies. The bacterial gene sequences at the junction with Tn5 were determined by using the forward and reverse Tn5-specific sequencing primers (supplied in the transposome kit). The bacterial sequences were then mapped against the genome sequence of *Enterobacter* sp. Ag1 [25] to identify where the Tn5 insertion was located.

### Complemented *waaL*^*c*^ strain

The *waaL*^−^ mutant was complemented with a plasmid with the *waaL* coding sequence. The entire *waaL* coding sequence was amplified with PCR using forward primer (5′-cagtctagaGATGGCCCTCACATTATTTTTTTC-3′) and reverse primer (5′-cagctcgagTTATTTTTTATTCAGTGCTATCAATAACC-3′). The restriction recognition sites Xba I (tctaga) and Xho I (ctcgag) were included in the forward and reverse primers, respectively. The plasmid pGFPuv was purchased from Clontech. The GFP gene in the plasmid was cut out with Xba I and Xho I, and replaced with the *waaL* PCR products by ligation. The mutant *waaL*^−^ cells were transformed with the engineered plasmids. The plasmids were extracted from transformants and verified for the presence of *waaL* by sequencing.

### Visualization of lipopolysaccharide on PAGE

LPS was extracted from the wt, *waaL*^−^ and complemented *waaL*^*c*^ using a kit (iNtRON Biotechnology, Bulldog Bio, NH) following the manufacturer’s instruction. The LPS preparations were separated on 12 % PAGE gels, and stained with LPS staining kit (Invitrogen, NY) following the manufacturer’s instruction. The LPS was visualized under UV light.

### Bacterial tagging

To be able to visualize bacteria in the mosquito gut, the wt, *waaL*^−^ and *waaL*^*c*^ bacteria were tagged with green fluorescent protein (GFP) or red fluorescent protein (RFP), respectively. The GFP carrying plasmid, pGFPuv was obtained from Clontech (Cat#: 632312). The plasmid was a pUC19 derivative pPD16.43 [26], the GFPuv gene was flanked by multiple cloning sites. To make a RFP carrier using the same plasmid, the GFPuv gene was excised from the plasmid using the enzymes XbaI and XhoI, and replaced with the RFP gene that was excised out from the pTurboRFP-B (Evrogen, Cat#: FP233). Therefore, the GFP and RFP genes were carried in the same plasmid. The strategy avoided potential issues that different plasmids might cause. The wt and mutant cells were transformed with pGFPuv and pRFP to generate strains wt/GFP, wt/RFP, *waaL*^−^/GFP, *waaL*^−^/RFP, *waaL*^*c*^/GFP and *waaL*^*c*^/RFP. Transformed bacteria were screened on plates for three generations, and stably tagged cells were used for the further experiments.

### Bacterial growth and co-culture of tagged bacteria

The cells of the wt, *waaL*^−^ mutant and complemented *waaL*^*c*^ strains were cultured overnight at 28 °C, and adjusted to OD_600nm_ of 0.1 in 20 mL LB medium, respectively, to initiate the cultures. The OD_600nm_ of each culture was measured hourly for 6 h. The growth curves were plotted for each strain. The experiments were conducted in triplicates. In co-culture assays, wt (GFP)/*waaL*^−^ (RFP), *waaL*^*c*^ (RFP)/*waaL*^−^ (GFP), wt (GFP)/*waaL*^*c*^ (RFP) were mixed in the ratio of 1:1, respectively, and grew in the same conditions as described above. The cultures were conducted in triplicates. A 5 μL of co-culture was placed on a glass slide with cover slip and examined with a fluorescent microscope (Nikon) to visualize the ratio of GFP and RFP tagged bacteria. The tagged bacteria were numerated respectively, the ratios of wt (GFP)/*waaL*^−^ (RFP), *waaL*^*c*^ (RFP)/*waaL*^−^ (GFP), and wt (GFP)/*waaL*^*c*^ (RFP) were analyzed by one way ANOVA, and Tukey’s method was used for *post hoc* comparison.

### Bacterial co-feeding assay

*Anopheles stephensi* was used for the study. The gut bacterial load in the newly emerged mosquitoes was very low, largely because the bacteria in the pupal gut are sequestrated during metamorphosis and are egested with meconium after emergence [27–29]. Therefore, we chose to feed bacteria to newly emerged mosquitoes to establish a desired bacterial community with dominance of introduced bacteria. Tagged bacteria were cultured in LB at 28 °C. Cells were collected at exponential phase and washed with 1× sterile PBS for three times. The combinations, wt/RFP and*waaL*^−^/GFP, *waaL*^−^/RFP and wt/GFP, wt/RFP and *waaL*^*c*^/GFP, *waaL*^−^/RFP and *waaL*^*c*^/GFP, were mixed in a ratio of 1:1 at OD_600nm_of 1.2 in a 10 % sucrose sugar solution, respectively. The bacterial quantity in the sugar solution was approximately 1 × 10^7^ colony forming unit (CFU)/mL. The bacteria were introduced into mosquitoes by oral feeding. Newly emerged mosquitoes were fed on tagged bacteria for 2 days. Then, the sugar-fed guts were dissected and observed using a fluorescent microscopy (Nikon) to visualize the bacteria. The remaining mosquitoes were given a blood meal. At 24 h post blood feeding, the blood-fed guts were dissected to visualize the bacteria.

### Mosquito infection with malaria

Three cohorts of newly emerged *An. stephensi* were used. One cohort was fed on regular 10 % sucrose solution as control mosquitoes. Second cohort was fed with the wt bacteria and the third cohort was fed with the mutant bacteria for 2 days, respectively. The three cohorts of mosquitoes were fed on the same mice infected with GFP-tagged *P.berghei*. Then the wt- and mutant-fed mosquitoes were maintained on sterile sugar meals until dissection. At day 7 post infected blood feeding, mosquito guts were dissected and examined under a florescent microscopy to visualize fluorescent oocysts. The infection pattern among three cohorts was compared by the infection rate (prevalence) and the parasite load per gut. Statistically, the difference of prevalence was tested by *χ*^2^, and the difference of oocyst number among three cohorts was compared using the ANOVA on ranks, and the *post hoc* was conducted by Dunn’s multiple comparison.

### Sensitivity to hydrogen peroxide

To test the sensitivity to H_2_O_2_, the wt and mutant cells were harvested in logarithmic phase. The cells were resuspended in 1× PBS and adjusted to OD_600nm_ of 0.2. The cells were exposed to 20 mM H_2_O_2_ or blank control for 30 min at 28 °C, respectively, and then plated on a LB plate and incubated at 28 °C overnight. The number of colonies was counted. The survival rates were calculated as colony counts of H_2_O_2_ exposed cells vs colony counts of blank control cells. The experiments were conducted in triplicates. The average of the survival rates between the wt and mutant were compared and the statistical significance was tested by one-way ANOVA and *post hoc* comparison was conducted by Donnett’s multiple comparison method.

### Ethics statement

This study was carried out under the NMSU and IACUC guidelines for biomedical research.

## Results

### The *waaL* disruption caused the lack of O-antigen in the LPS structure

**Fig. 1:**
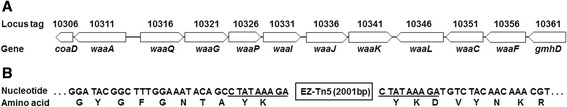
Genomic location of *waaL* and Tn5 insertion in *waaL*. **a** The genomic region where a cluster of *waa* genes are located. The locus tag (the prefix A936_ was omitted) and gene names were indicated. The *waaL* gene encodes a 402 aa protein. The *waaL* gene was *boxed*. **b** Insertion site of the Ez-Tn5 transposon in the *waaL* gene. The transposon was inserted between the 2nd (A) and 3rd (T) nucleotide of the 298th codon (GAT/D) and generated a 9 bp direct repeat (*underlined*) flanking the Tn5 insert

A mutant library of *Enterobacter* sp. Ag1 was generated using an EZ-Tn5 mutagenesis kit. A mutant was identified with the disruption of gene *waaL* by the transposon Tn5 insertion. The *waaL* gene codes for O antigen ligase. Structurally, LPS is comprised of three regions: the lipid anchor (lipid A), the inner and outer core oligosaccharide (OS), and the O-antigenic polysaccharide (O-PS) [30]. The O antigen ligase is responsible for the ligation of O antigen to the outer core of LPS [31]. The *waaL* gene is located in a locus harboring a cluster of genes responsible for the LPS core oligosaccharide biosynthesis and assembly [30]. Figure 1a presents the gene cluster in the genome of *Enterobacter* sp. Ag1 [25], and Fig. 1b depicts the Tn5 insertion of *waaL* in the mutant.

**Fig. 2:**
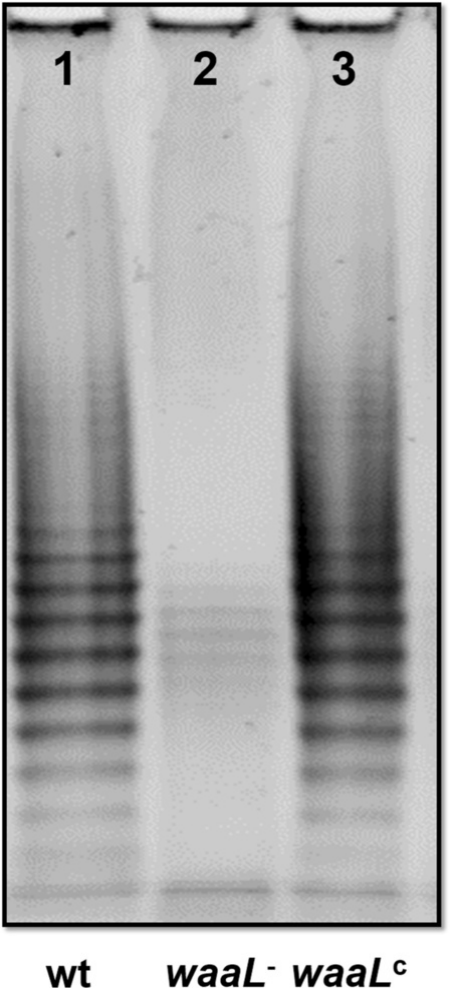
LPS pattern on a 12 % PAGE gel. Compared to the wt (*1*), O antigen ladder was missing in the *waaL* ^−^ (*2*) and in the complemented strain *waaL* ^*c*^ (*3*), the O antigen ladder was restored

**Fig. 3:**
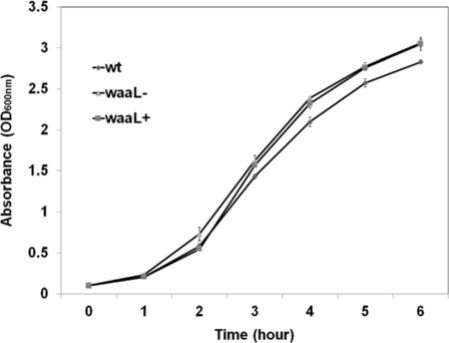
Growth pattern of the wt, *waaL* ^−^ and *waaL* ^*c*^. Bacteria were cultured in the LB medium. The absorbance of OD_600nm_ was measured hourly. The data were presented with mean ± SD of absorbance from three replicates

The *waaL*^−^ mutation caused a defect in LPS structure. On a PAGE gel, the LPS of the wt strain displayed a typical ladder-like banding pattern with varying lengths of O-PS repeats (Fig. 2, lane 1). The LPS ladder was almost missing in the *waaL*^−^ (Fig. 2, lane 2). When the *waaL*^−^ was complemented with a plasmid carrying a full-length of *waaL*, the O-PS repeats were restored (Fig. 2, lane 3). This was consistent with the function of O-antigen ligase, mediating the attachment of O-PS to the core of LPS. Interestingly, the lack of O-PS appears not to affect growth *in vitro*. The cultures of three strains, wt, *waaL*^−^ and *waaL*^*c*^, showed very similar growth curves (Fig. 3).

### The *waaL*^−^ was less competitive than wt both in the gut and in the co-culture *in vitro*

**Fig. 4:**
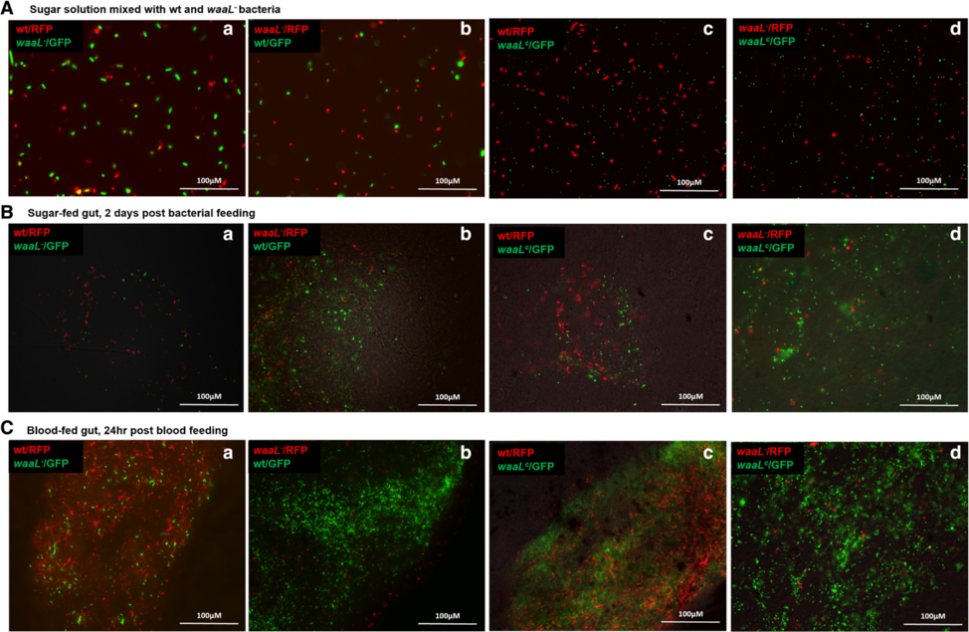
Co-feeding assay with GFP or RFP tagged bacteria. Newly emerged mosquitoes were fed on sugar meals mixed with wt/RFP and *waaL* ^−^/GFP (*a*), *waaL* ^−^/RFP and wt/GFP (*b*), wt^−^/RFP and *waaL* ^*c*^ /GFP (*c*) or *waaL* ^−^/RFP and *waaL* ^*c*^/GFP (*d*) cells in 1:1 ratio for 2 days. Then, the bacterial sugar was replaced with sterile sugar solution for the remaining time of the experiments. At day 3, the mosquitoes were given a blood meal. Panel (**a**) presents the fluorescent bacteria in the sugar solution; Panel (**b**) presents the fluorescent bacteria in the gut examined at day 3 post bacterial feeding; Panel (**c**) presents the fluorescent bacteria in the gut at 24 h post blood feeding

*Enterobacter* is one of the predominant inhabitants in the mosquito gut [7]. To test if the lack of O-PS affects the inhabitance of bacteria in the mosquito gut, fluorescently tagged bacteria were used to track their dynamics in the mosquito gut. Preliminary results showed that the number of ingested wt bacteria varied in different individual guts over time. In addition, it was unfeasible to accurately quantify all tagged bacteria in a gut using gut images. Therefore, we developed a co-feeding assay to examine the colonization capability of the *waaL*^−^. The bacteria were tagged with GFP and RFP, respectively. These combinations were used for co-feeding study, wt/RFP and *waaL*^−^/GFP, *waaL*^−^/RFP and wt/GFP, wt/RFP and *waaL*^*c*^/GFP, *waaL*^−^/RFP and *waaL*^*c*^/GFP. Tagged bacteria were mixed in a ratio of 1:1 in sugar meals (Fig. 4A). Newly emerged *An. stephensi* were fed with tagged bacteria for 2 days. On the day 3 post bacterial feeding, the mosquito guts were examined for the bacterial status. As shown in Fig. 4B, the wt and complemented bacteria steadily populated in the sugar-fed gut, but *waaL*^−^ bacteria were less seen in the gut. The mosquitoes were then given a blood meal. The gut bacteria were examined at 24 h post blood feeding. As shown in Fig. 4C, the mutants were much less prevalent than the wt and complemented bacteria in the blood-fed gut. Apparently, in the co-feeding assay, the mutant cells were less competitive than the wt and complemented cells in the gut environment. Since the mutant cells grew normally in LB culture (Fig. 3), we used a co-culture assay to test if the growth would change in the presence of wt cells. Indeed, the mutant cells grew at a slower rate than the wt cells. As shown in Table 1, the ratios of both wt vs *waaL*^−^ and *waaL*^*c*^ vs *waaL*^−^ were around 1 in the initial of culture, then became significantly higher at 6 h and/or 12 h (One way ANOVA, *P* < 0.05, *post hoc* with Tukey’s multiple comparisons test). The complementation restored the growth to normal pattern, as the *waaL*^*c*^ cells showed similar growth rate as the wt cells (Table 1). Like the pattern in the gut, the mutant cells were less competitive in the co-culture assay as well. Taken together, the lack of O-PS did not affect bacterial proliferation when growing alone in culture (Fig. 3) but did compromise the competency to compete with other cells both *in vitro* (Table 1) and *in vivo* (Fig. 4).

**Table 1:** The growth ratio of wt/mutant in the co-culture

Co-culture	Ratio (mean ± SD)^a^		
	0 h	6 h	12 h
wt (GFP)/ *waaL* ^−^ (RFP)	1.19 ± 0.07^a^	3.64 ± 0.92^ab^	5.24 ± 1.77^b^
*waaL* ^*c*^ (RFP)/ *waaL* ^−^ (GFP)	1.34 ± 0.01^a^	4.4 ± 1.38^b^	6.85 ± 0.64^b^
wt (GFP)/ *waaL* ^*c*^ (RFP)	0.98 ± 0.12^a^	1.29 ± 0.07^a^	1.8 ± 0.65^a^

^a^The numbers of fluorescent cell in a co-culture were numerated at 0, 6 and 12 h and ratios were calculated. The ratios in each co-culture were analyzed using one way ANOVA. Tukey test was used as *post hoc* comparison. Significant difference was indicated by different letters.

### The mutant bacteria were vulnerable in the presence of blood *in vitro*

*Enterobacter* thrived in the blood fed gut. The LPS deficiency may render mutants more vulnerable to the innate immune factors in the vertebrate blood. To examine the effect of blood on the mutant strain, the cells were incubated in heparinized mouse blood for 1 and 3 h, respectively. Cells were then spread on LB plates with appropriate dilutions. The cell viability was measured by CFU on the plates. At 1 h post blood incubation, the CFU increased 2.6 ± 0.5 times in wt, 2.3 ± 0.2 times in *waaL*^*c*^, and 2.0 ± 0.4 times in *waaL*^−^, compared to the CFU at initial time point (0 h), there was no significant difference among them (One way ANOVA, *P* > 0.05). However, at 3 h post incubation, the CFU of wt and *waaL*^*c*^ increased 13.4 ± 1.7 and 8.8 ± 1.4 times, respectively, while the CFU increased only 7.5 ± 0.9 times in *waaL*^−^ (One way ANOVA, *P* < 0.05). The viability of *waaL*^−^ was lower than that of the wt, suggesting that O-PS structure may be involved in the protection from harmful factors in the blood. The CFU of *waaL*^*c*^ were more than that of *waaL*^−^, but the difference was not significant (*P* > 0.05).

### The *waaL* mutants were more sensitive to H_2_O_2_

The *waaL*^−^ mutant was obtained by screening the mutant library with paraquat, an oxidative stressor (see Methods). In line with reports in the literature, the *waaL* mutants in *Edwardsiella tarda* [32], *Pseudomonas aeruginosa* and *Erwinia amylovora* [33] showed higher sensitivity to oxidative stress. Therefore the effect of *waaL* mutation on the vulnerability to oxidative stress was examined. The sensitivity to H_2_O_2_ was compared for the wt, *waaL*^−^ and *waaL*^*c*^. The cells were exposed to 20 mM H_2_O_2_ or blank control for 30 min, respectively, and then were spread on LB plates. As shown in Table 2, the *waaL*^−^ had a lower survival rate (39 %) when compared with the wt (79 %, One-way ANOVA, *P* < 0.01, Dunnett’s comparison versus the wt, *P* < 0.05). The complementation of *waaL*^*c*^ partially restored the protection, the survival rate (59 %) was close to that of the wt (Dunnett’s comparison, *P* > 0.05).

**Table 2:** Sensitivity to hydrogen peroxide

	Cell counts and survival rates (mean ± SD)		
	wt	*waaL* ^−^	*waaL* ^*c*^
H_2_O_2_ absent	856.3 ± 51.4	685.0 ± 91.0	758.0 ± 69.4
H_2_O_2_ present	674.3 ± 111.6	269.0 ± 91.9	443.7 ± 19.1
Survival rate	0.79 ± 0.16	0.39 ± 0.08	0.59 ± 0.06

The cell counts post exposure to 20 mM H_2_O_2_ were obtained from three replicates. The survival rates were analyzed by one way ANOVA (*P* < 0.01). Dunnett’s method was used for comparisons, *waaL* ^−^ vs wt, *P* < 0.05; *waaL* ^*c*^ vs wt, *P* > 0.05

### The mutant-fed mosquitoes were more susceptible to rodent malaria

**Fig. 5:**
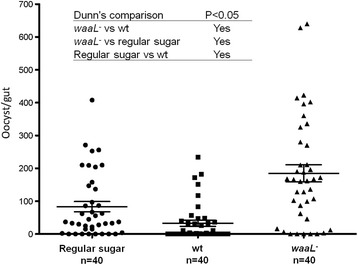
The effect of *Enterobacter* strains on mosquito susceptibility to malaria *P. berghei* infection. The parasite loads of each individual in the three cohorts were presented. The mean ± SD of oocysts was indicated by the horizontal line. The parasite loads were significantly different among the three cohorts (ANOVA on rank, *P* < 0.01). Dunn’s comparisons between the cohorts were presented in the insert panel.

Some species of *Enterobacter* have been shown to be involved in the mosquito immunity against malaria [6, 16, 24]. To determine if the *waaL* mutation affect mosquito susceptibility to rodent malaria *Plasmodium berghei*, malarial infection patterns were compared in the mosquitoes fed on the wt and *waaL*^−^ mutants. Mosquitoes were set as three cohorts. One cohort was fed on the regular sugar as a control. The other two cohorts were fed on sugar supplemented with the wt bacteria and the *waaL*^−^ bacteria, respectively. The three cohorts were then fed on the same set of mice infected with *P. berghei*. The malaria infection was assessed by the prevalence and the oocyst number per gut. As shown in Fig. 5, the control mosquitoes that were fed on regular sugar showed prevalence of 80 % and average parasite load of 83.5 oocyst/gut. The wt-fed mosquitoes showed reduced prevalence of infection (52.5 %) and less parasite load (mean = 33 oocyst/gut), while the mutant-fed mosquitoes had higher infection (mean = 184.9 oocyst/gut, 87.5 % of prevalence). The prevalence of infection between the wt and mutant was significantly different (*χ*^2^ test, *P* < 0.01), and the oocyst load among three cohorts was statistically different as well (Kruskal-Wallis ANOVA on ranks, *P* < 0.01, Dunn’s all pairwise multiple comparison procedures, *P* < 0.05). In a replicate experiment, the same pattern was observed (data not shown).

## Discussion

Transposon mediated mutagenesis has been widely used in bacterial gene function study [34–36]. In this study, we generated a Tn5 mediated mutant library of *Enterobacter* sp. Ag1. The *waaL*^−^ mutant was characterized. The disruption of the *waaL* abolished the attachment of O-PS to the outer core of LPS, which resulted in the O-PS deficiency. The LPS in the wt bacteria displayed a ladder of O-PS repeat units on a PAGE gel, a typical pattern of smooth type of LPS. The banding pattern of O-PS repeats was absent in the *waaL*^−^ mutant, the pattern was restored by complementation as expected (Fig. 2), indicating the requirement of *waaL* in the O-PS attachment during LPS synthesis in *Enterobacter* sp. Ag1. There are two types of LPS in Gram negative cells, the smooth type that O-PS is present and the rough type that O-PS is absent [37].

The LPS defect appeared not to interfere with the growth when the mutant was alone in the LB culture. The *waaL*^−^ grew in a rate similar to the wt and complemented bacteria (Fig. 3). However, when growing together with the wt cells in the co-culture assay, the mutant cells grew at a slower rate (Table 1). A similar pattern was observed *in vivo*. In the co-feeding assay, after being ingested via sugar meals, the wt bacteria steadily populated in the gut, while the *waaL*^−^ bacteria were less prevalent in both sugar-fed and blood-fed guts (Fig. 4). These findings suggest that the absence of O-antigen compromised the competency of proliferation in co-culture *in vitro* and co-feeding assays *in vivo*, although the conditions in LB culture and in the gut are quite different. Interestingly, the O-antigen has been associated with the social gliding mobility (cells move as groups) in *Myxococcus xanthus* [38] and social swarming mobility in *Pectobactrium atrosepticum* [39]. In a community setting, cell-cell interactions may be essential for bacterial growth . The lack of O-antigen in the mutants made it less competent in proliferation in both co-culture and co-feeding assays (Table 1, Fig. 4), suggesting the O-antigen may be involved in cell-cell interactions in a microbial community setting.

In the blood-fed gut, more complicated situations could happen. The mammalian blood contains hostile immune factors such as antibodies and complement. It has been documented that human complement was active in the gut for 1 hour after ingestion [40], the *Aedes aegypti* mosquitoes produced complement system inhibitors to protect gut epithelial cells from possible injury by the complement [41]. These mammalian immune factors may adversely affect bacteria in the gut. It has been shown that serum antibodies were able to mediate complement-dependent killing of various bacteria [42, 43]. In some cases, the O-antigen could confer bacteria resistance to host immune defenses especially to complement protein deposition and complement lytic activity [44, 45]. In *Bordetella parapertussis* O antigen dependent protection against complement-mediated control and clearance was necessary for the bacteria to efficiently colonize the lower respiratory tract [45]. In our case, the *waaL*^−^ was less viable at 3 h post incubation with the blood *in vitro*. It is conceivable that mammalian host immune factors in a blood meal may contribute to the competition defect in the blood-fed gut in the co-feeding assay. Taken together, the *waaL*^−^ defect in competition either *in vitro* or *in vivo* may associate with different mechanisms in different scenarios. Further studies are necessary to characterize these context dependent mechanisms. In the squid-*Vibrio* symbiotic system, the LPS structure, especially O-antigen, is required for *Vibrio fischeri* to colonize the squid light organ [46]. Similarly, the O-antigen-deficiency in *Salmonella enterica* serovar typhimurium compromised its colonization in the Peyer’s patches and liver of mice [47]. In *Rhizobium tropici* CIAT899 LPS defects impaired maize rhizosphere and root colonization [48]. Recently, it was shown that a phosphatase mediated lipid A modulation was required for the periodontal colonization of *Porphyromonas gingivalis* in a rabbit model [49]. These data exemplified solid connections between LPS structure and host colonization in various circumstances.

The *waaL*^−^ mutant was selected by paraquat screening. It was sensitive to H_2_O_2_ as well (Table 2)_._ The results were reminiscent of the phenotypes of *waaL* mutants of *Erwinia amylovora*, *Pseudomonas aeruginosa* [33] and *Edwardsiella tarda* [32]. The *waaL* mutation in these species resulted in higher sensitivity to H_2_O_2_. At the present, it is not clear how the LPS deficiency impairs the defense against oxidative stress in the *waaL*^−^ mutant. A possible explanation is that defective LPS may render the outer membrane less protective, which may increase permeability to oxidants. The O-antigen dependent protection against oxidative stress may be of ecological significance for *Enterobacter* to thrive in the blood-fed gut [7]. Blood feeding makes mosquito gut a stressful environment due to the elevated oxidative stress that is associated with the hemoglobin digestion and metabolism [50]. The bacteria and ingested blood are confined by a transient peritrophic matrix that is produced by mosquito gut cells to spatially separate the blood bolus from the gut epithelia [51]. Therefore, the gut bacteria are directly exposed to the oxidative stress derived from blood digestion. We have shown that blood feeding shifts microbial structure in the mosquito gut by reducing the taxonomic diversity and increasing the abundance of bacteria in the family Enterobacteriaceae [7]. In the blood-fed gut, the LPS dependent stress defense may be one of the protections for bacteria to thrive in the stressful environment. The impaired defense against oxidative stress in the *waaL*^−^ may contribute to the competition defect in the blood-fed gut. In maintaining a healthy host microbial symbiosis, redox homeostasis plays a pivotal role in cross-talks among different parties in a host associated metagenomic ecosystem [52]. The findings in the current study suggest that an intact LPS structure is critical for *Enterobacter* to reside stably in the mosquito gut by several different means possibly from protections to cell-cell interactions.

The gut microbial community plays an essential role in maintaining a basal immunity in mosquitoes [11, 22]. In the family Enterobacteriaceae, some strains of *Enterobacter* [16, 24, 53] and *Serratia* [54] have been shown to be influential in malaria susceptibility. In line with those observations, wt-fed mosquitoes had lower *P. berghei* infection, while the *waaL*^−^-fed mosquitoes showed higher infection (Fig. 5). The data suggest that the wt *Enterobacter* could populate the gut well and promoted stronger immunity against malaria. This effect was compromised in the mutants, likely due to the less competitiveness in the gut community (Fig. 4). Alternatively, LPS may be immunogenic to prime basal immunity in the gut. It is worth noticing that the *waaL* gene of *Aeromonas hydrophila* was required for stimulation of melanization in the insect, *Tenebrio molitor* [55], and in *Maduca sexta* the LPS with O-antigen was more potent than the lipid A and rough mutants of LPS in activation of antimicrobial gene expression [56], although LPS does not activate *Drosophila* immune responses in adults [57, 58]. Further study is required to determine if LPS is involved in the activation of anti-malaria immunity. The availability of LPS deficient mutants enables one to study the roles of LPS structure of *Enterobacter* in the symbiotic relationship with the mosquito host. Further investigation is underway to characterize the dynamics of *Enterobacter* in the mosquito gut and the impact on the mosquito traits, such as fecundity and gut local immunity.

## Conclusions

In the mosquito gut, *Enterobacter* is a predominant resident in the microbial community. Lack of the O antigen structure in LPS of *Enterobacter* compromised its effective growth in co-culture and reduced its prevalence in the co-feeding assays. In addition, O-antigen deficiency increased the sensitivity to oxidative stress. The findings suggest intact LPS is required for the bacteria to steadily stay in the gut.

## Abbreviations

LPS: Lipopolysaccharide
CFU: Colony forming unit
GFP: Green fluorescent protein
RFP: Red fluorescent protein
KAN: Kanamycin
